# Levels of sdRNAs in cytoplasm and their association with ribosomes are dependent upon stress conditions but independent from snoRNA expression

**DOI:** 10.1038/s41598-019-54924-2

**Published:** 2019-12-05

**Authors:** Anna M. Mleczko, Piotr Machtel, Mateusz Walkowiak, Anna Wasilewska, Piotr J. Pietras, Kamilla Bąkowska-Żywicka

**Affiliations:** 1 Institute of Bioorganic Chemistry Polish Academy of Sciences, Noskowskiego 12/14, 61-704 Poznań, Poland; 2 Centre for Advanced Technologies, Poznań, Poland

**Keywords:** Small RNAs, Ribosome

## Abstract

In recent years, a number of small RNA molecules derived from snoRNAs have been observed. Findings concerning the functions of snoRNA-derived small RNAs (sdRNAs) in cells are limited primarily to their involvement in microRNA pathways. However, similar molecules have been observed in *Saccharomyces cerevisiae*, which is an organism lacking miRNA machinery. Here we examined the subcellular localization of sdRNAs in yeast. Our findings reveal that both sdRNAs and their precursors, snoRNAs, are present in the cytoplasm at levels dependent upon stress conditions. Moreover, both sdRNAs and snoRNAs may interact with translating ribosomes in a stress-dependent manner. Likely consequential to their ribosome association and protein synthesis suppression features, yeast sdRNAs may exert inhibitory activity on translation. Observed levels of sdRNAs and snoRNAs in the cytoplasm and their apparent presence in the ribosomal fractions suggest independent regulation of these molecules by yet unknown factors.

## Introduction

snoRNAs are noncoding RNAs that contribute to ribosome biogenesis and RNA splicing by modifying ribosomal RNA and spliceosomal RNAs, respectively. However, recently emerging evidence suggests that some snoRNAs have non-canonical functions in RNA editing, alternative splicing or maintenance of chromatin structure^1–3^. The mechanistic details of these non-canonical functions are largely unknown. Moreover, it has recently become evident that mature, functional snoRNAs undergo processing to form stable short fragments, termed psnoRNAs, for processed snoRNAs^4^ or sdRNAs for snoRNA-derived RNAs^5^. The presence of processed forms of snoRNAs has been demonstrated in several organisms, including the primitive protozoan *Giardia lamblia*^6^, Epstein-Barr virus^7^, the budding yeast *Saccharomyces cerevisiae*^8^, and mammals^4,9–13^.

Concerning possible functions of small RNAs derived from snoRNAs, their potential to regulate alternative splicing events^4^ as well as their microRNA–like abilities, have been described in several organisms^10,14,15^. Recently, it has been postulated that FUS-dependent sdRNAs in human cell lines might regulate gene expression, affecting transcript stability and translation^16^. As the majority of miRNA-targeted, and thus translationally repressed, mRNAs are distributed in the cytoplasm, miRNA-like sdRNAs are expected to co-localize within the cytoplasm. Indeed, a subset of small RNAs derived from snoRNAs have been detected in the cytoplasm in *G*. *lamblia*^14^ and humans^10^; however, their nucleolar localization has been reported as well^11^. Our recent work presented another possibility of sdRNA localization within the cytoplasm by association with ribosomes^8^. These studies were performed in *S*. *cerevisiae*, an organism lacking miRNA machinery; hence, one might suspect a distinct role for non-miRNA sdRNAs in yeast.

It is now commonly accepted that full-length snoRNAs are not exclusively localized within the nucleus but are also present in the cytoplasm. Moreover, their cytoplasmic abundance is dynamically regulated in various stress conditions, such as oxidative stress^17^, lipotoxic conditions^18^ or heat shock^19^. Knowledge concerning snoRNA expression regulation, however, is sparse. In 2002, it was shown for the first time that snoRNAs are involved in cancer development^20^. Since that time, a growing body of evidence has emerged linking both full-length snoRNAs and their derivatives to oncogenesis (reviewed in^21^). Considering both localization and the potential functions of full-length snoRNAs and sdRNAs, one might suspect that snoRNAs and their derivatives orchestrate responses to environmental stress outside of the nucleus. Surprisingly, studies aimed at revealing the abundance and subcellular localization of sdRNAs in stress have yet to be reported.

Yeast sdRNAs are observed in limited numbers during the sequencing of ribosome-associated small RNAs, with a maximum of 15 copies in the rancRNA library^8^. However, their regulatory potential cannot be excluded, as recent studies demonstrated that even relatively small levels (when compared to ∼200,000 ribosomes/cell) of ribosome-associated noncoding RNA (ranc_18mer, ∼27,000 molecules/cell) are sufficient to substantially influence global ribosome activity and regulate translation^22^. We recently demonstrated that due to sdRNA low abundance, conventional techniques, such as northern hybridization, are not sensitive enough to detect the full repertoire of cellular sdRNAs^23^. Poor sensitivity and low throughput of hybridization-based technologies can be overcome by sensitive, amplification-based detection methods, such as stem–loop reverse transcription PCR (SL-RT-PCR), originally described by Chen *et al*.^24^ and successfully implemented by our group for detection of yeast sdRNAs from as little as 50 ng of low molecular weight cellular RNA^23^.

Therefore, to investigate the subcellular localization of both full-length snoRNAs and snoRNA-derived small RNAs in *S*. *cerevisiae*, we used this amplification-based method. To enable absolute quantification of RNAs, we implemented digital droplet PCR (ddPCR) technology. Our comprehensive analysis of sdRNA and snoRNA abundance and localization was performed under 12 different yeast growth conditions. Herein, we present the first evidence that snoRNA levels and the localization of sdRNAs within the cell, including association with ribosomes, are dependent upon stress conditions. As a consequence of sdRNA binding to ribosomes, an inhibition of *in vitro* and *in vivo* translation occurs. Moreover, for the first time, we present experimental data suggesting that both the expression and ribosome association of two types of related RNAs, namely, snoRNA-derived small RNAs and full-length snoRNAs, are independent from each other during multiple growth conditions.

## Materials and Methods

### snoRNA-derived small RNAs

**Figure 1:**
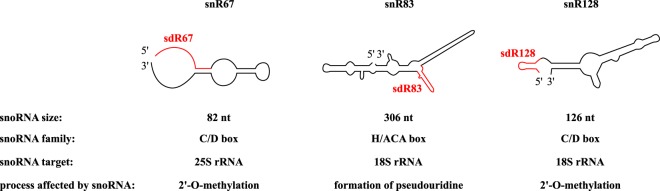
snoRNAs and sdRNAs used in this study. Localization of sdRNAs within predicted snoRNAs secondary structures and functional details of snoRNAs is shown.

Three snoRNAs (snR67, snR83 and snR128) and 3 corresponding sdRNAs (sdR67, sdR83 and sdR128) were chosen for analysis based on the highest read coverage observed in ribosome-associated small RNA sequencing in *S*. *cerevisiae*^8^. Sequences of sdRNAs are shown in Table 1. Localization of sdRNAs within predicted snoRNA secondary structure is shown in Fig. 1.

**Table 1:** Oligonucleotide sequences used in this study.

Name	Type	Sequence [5′-3′]	Length [nt]
snR67	Fwd primer	TAACATGATGACTAAGTTGTCGCC	24
	RT primer	TTTCAGAATTTTCAGTGTTTGTTGTTTG	28
sdR67	**RNA**	**AACAUGAUGACUAAGUUGU**	**19**
	RT primer	GTCGTATCCAGTGCAGGGTCCGAGGTATTCGCACTGGATACGACACAACT	50
	Fwd primer	GGCGCGCGCGAACATGATGACTA	23
snR83	Fwd primer	CCCAAAAACATCAAGAAAAGCCTTT	25
	RT primer	AACTGTCGCCCTTAATATTAGTCCC	25
sdR83	**RNA**	**GGACCAAUUACCGUAGUUGCGACUACAACAAUUUUGUUCAUA**	**42**
	RT primer	GTCGTATCCAGTGCAGGGTCCGAGGTATTCGCACTGGATACGACTATGAA	50
	Fwd primer	GACCAAUUACCGUAGUUGCGAC	22
snR128	Fwd primer	TCACGGTGATGAAAGACTGGT	21
	RT primer	TCACTCAGACATCCTAGGAAGGT	23
sdR128	**RNA**	**CACGGUGAUGAAAGACUGGUU**	**21**
	RT primer	GTCGTATCCAGTGCAGGGTCCGAGGTATTCGCACTGGATACGACAACCAG	50
Spike-in	**RNA**	**AUAGGCCAUAAGGAGUCUCGGUACGUCUUGUAUG**	**44**
	RT primer	GTCGTATCCAGTGCAGGGTCCGAGGTATTCGCACTGGATACGACCATACA	50
	Uni primer	CCAGTGCAGGGTCCGAGGTA	20

sdRNA sequences are presented in bold. RT primer – primer used for reverse transcription; Fwd primer – forward ddPCR primer; Uni primer – universal reverse ddPCR primer.

### Strain and growth conditions

*Saccharomyces cerevisiae* strain BY4741 was grown in YPD medium with 2% glucose at 30 °C. Environmental stress was induced as previously described^25^ in two biological replicates. Briefly, cells were grown to mid-log phase, and stress conditions were applied for 15 min. Next, cells were pelleted and stored at −20 °C. Stress conditions were as follows: heat shock (37 °C), cold shock (15 °C), high salt conditions (1 M NaCl), high pH conditions (pH 7.9), low pH conditions (pH 4.0), UV exposure (120 J/m^2^ UV), hyperosmotic shock (1 M sorbitol), hypoosmotic shock (cells grown to mid-log phase in YPD supplemented with 1 M sorbitol were transferred to YPD without sorbitol), amino acid starvation, sugar starvation, and anaerobic and normal growth.

### Yeast lysates and ribosome preparation

Yeast ribosomes were prepared as previously described^26,27^. Briefly, cell pellets were washed with ice-cold water and resuspended in buffer (10 mM MgCl_2_, 100 mM KCl, 50 mM Tris/HCl, pH 7.5, 0.4 mM PMSF) at 4 °C. Equal volumes of glass beads (400 μm in diameter) were added, and cells were broken using 8 pulses of vortexing (30 sec each) punctuated by cooling on ice. Cell debris was precipitated at 11,300 × g for 2 min at 4 °C in F-34-6-38 Eppendorf rotor. Lysate was further clarified by centrifugation at 11.300 × g for 10 min at 4 °C in F-34-6-38 Eppendorf rotor. After clarification, 1/10 of the total lysate volume was used to isolate total cellular RNA (S30). Subsequently, ribosomes were pelleted (P100) from lysates by centrifugation at 160,000 × g for 90 min at 4 °C in Beckman 70.1 Ti rotor and suspended in the storage buffer (2 mM Mg(OAc)_2_, 100 mM KOAc, 20 mM HEPES, pH 7.4, 0.1 mM PMSF, 1 mM DTT, 20% glycerol). The top two-thirds of the post-ribosomal supernatant were collected and frozen, and designated as the S100 fraction. P100, S100 and S30 fractions were mixed with TRI Reagent (MRC), flash frozen in liquid nitrogen and subjected to RNA isolation following the manufacturer’s instructions. The purity of P100 and S100 fraction was verified with Agilent Bioanalyzer 2100 with the use of RNA Nano 6000 kit.

### Reverse transcription

Stem-loop RT primers for sdRNA amplification (Table 1) were designed as previously described^23^. Standard RT primers for snoRNA amplification were designed using the Primer3Plus tool. All reverse transcription reactions were performed in a multiplex manner. Reverse transcription reactions contained 10 or 100 ng RNA from P100, S100 or S30 fractions, 50 nM of each stem-loop RT primer for sdRNAs and spike-in RNA, 50 nM of each standard RT primer for snoRNAs, 1 × RT buffer, 0.25 mM of each dNTPs, 50 U SuperScript SSIII reverse transcriptase (Invitrogen), 5 U RiboLock RNase Inhibitor (Thermo Scientific), 10 mM DTT and 500 fM spike-in RNA (Table 1) as a normalizer. Twenty-microlitre reactions were incubated in a Bio-Rad T100TM Thermocycler for 30 min at 16 °C, followed by pulsed RT of 60 cycles at 30 °C for 30 sec, 42 °C for 30 sec, and 50 °C for 1 sec.

### Digital droplet PCR (ddPCR)

Copy numbers of sdRNAs and snoRNAs were determined using the QX100™ Droplet Digital™ PCR system (Bio-Rad, Pleasanton, CA). The reaction mixture was composed of 10 µl of 2x QX200™ ddPCR™ EvaGreen Supermix, 200 nM specific forward and universal reverse primers (Table 1), and 1 µl cDNA.

### Translation of poly(U) templates *in vitro*

Translation of poly(U) templates was performed as described^28^ using 5 A260 units of ribosomes isolated from yeast grown under optimal and stress conditions, 25 mg poly(U), 100 mg soluble protein factors isolated from yeast grown under optimal and stress conditions, 25 mg deacylated yeast tRNA and 0.3 nmol [^3^H]-phenylalanine. The reaction was performed at 30 °C for 30 min. Products were precipitated in TCA, recovered on Whatman glass fibre GF/C filters and subjected to scintillation counting. *In vitro* translation assays were performed in triplicate. Reported values are corrected for control samples lacking ribosomes, which were typically 0.5% to 1% of the total probe counts applied.

### *In vitro* translation

*S*. *cerevisiae* cell-free extracts were prepared in the cold-room, as previously described in^29^ with modifications. To prepare *S*. *cerevisiae* S30 extract, yeast culture was grown to a final OD600 of 1.2 at 30 °C in YPD medium. Cells were chilled on ice, harvested by centrifugation at 1,500 × g for 5 min at 4 °C in F-34-6-38 Eppendorf rotor and washed five times with 30 ml of ice-cold buffer A (30 mM HEPES/KOH, pH 7.6, 100 mM KOAc, 3 mM Mg(OAc)_2_, 2 mM DDT, 0.5 mM PMSF) supplemented with 8.5% (w/v) mannitol. Subsequently, cell pellet was resuspended in 1.5 ml of buffer A (supplemented with 8.5% mannitol and 0.5 mM PMSF) per 1 g of the cell pellet and six-time weight of cold glass beads (400 µm in diameter) was added.

Cells were broken by performing eigh-1 min cycles of vortexing (30 sec) and handshaking (30 sec, approximately 2 Hz over 50 cm hand patch). To remove the glass beads, the lysates were centrifuged at 120 × g for 2 min at 4 °C in F-34-6-38 Eppendorf rotor, transferred to a fresh tube, and centrifuged at 30,000 × g for 7 min at 4 °C in Hettich ROTINA 380 R rotor. The resulting S30 supernatant was purified on a G-25 Sephadex column. A portion of purified S30 extract was supplemented with 1 mM CaCl_2_ and treated with 50 U/ml micrococcal nuclease at 26 °C for 10 min in order to eliminate endogenous mRNAs. The reaction was stopped by adding 2.5 mM EGTA. Both types of S30 extracts were aliquoted, snap-frozen in liquid nitrogen and stored at −80 °C.

For *in vitro* translation assays, 4 µl master mix (50 mM HEPES/KOH, pH 7.6, 2.5 mM ATP, 250 µM GTP, 50 mM creatine phosphate, 5 mM DTT, 0.6 U creatine phosphokinase, 125 mM KOAc, 5 mM MgOAc, 25 µM amino acid mix (-Met), 4 U Ribonuclease Inhibitor, 500 nCi ^35^S-methionine (1,000 Ci/mmol, 10 mCi/ml)) was mixed with 5 µl cell-free extract. Synthetic sdRNA or control RNA oligomers (10–500 pmol) or cycloheximide (7.5 μg/μl) was added to reach a final volume of 14 μl and mixtures were incubated at 26 °C for 40 min. The labeled proteins were precipitated by adding four volumes of ice-cold acetone and incubating at −20 °C for 30 min. Then, samples were centrifuged at 16 300 × g for 15 min at 4 °C in Eppendorf FA-45-24-11 rotor. The precipitate was dissolved in 15 µl 1x loading buffer (50 mM Tris-Cl, pH 6.8, 2% (w/v) SDS, 0.1% (w/v) bromophenol blue, 10% (v/v) glycerol, 100 mM DTT). Proteins were resolved on 10% SDS-polyacrylamide gels using 1xTGB running buffer and visualized on phosphor – storage intensity screen (Fujifilm) overnight. Screens were scanned using Fujifilm Fluorescent Image Analyzer FLA – 5100. We have measured the intensity of labelled proteins within the gel and subtracted background screen intensity. All measurements were performed at least in triplicate and standard error (SE) has been calculated. p-value has been calculated using t-test.

*In vitro* translation reactions were performed in wheat germ (Promega), rabbit reticulocyte (Promega), and human (Pierce Human *In Vitro* Protein Expression Kit, Fisher Scientific) systems according to the manufacturers’ instructions. The reactions were incubated for 90 min. at 25 °C (wheat germ extract) or at 30 °C (rabbit reticulocyte and human systems). In all cases, an uncapped *in vitro*-transcribed luciferase mRNA containing a 30-base poly(A) tail was used as a template. ^35^S-labeled proteins were resolved using SDS-PAGE and visualized on a storage phosphor intensity screen (Fujifilm) overnight. Screens were scanned using a Fujifilm Fluorescent Image Analyzer FLA-5100. All reactions were performed in triplicate and standard error (SE) has been calculated.

### Metabolic labeling

Yeast spheroplasts were prepared from a 50-ml culture grown to an OD600 of 0.8 by adding 350 U of zymolyase (Zymo Research) and incubating at 30 °C for 25–30 min as previously described^22^. Spheroplasts were combined with synthetic sdRNA (10–100 pmol) or 100 pmol control RNA oligomers (scr-sdR128 5′-CUUGAGAUGAUUGCUAUGAUAC-3′, scr-ranc18 5′-AAGUGAAGAAGGAAGAAA-3′ or spike-in RNA 5′-AUAGGCCAUAAGGAGUCUCGGUACGUCUUGUAUG-3′) and electroporated. For controls, translation was inhibited by adding 7.5 μg/μl cycloheximide to the spheroplasts. Electroporated spheroplasts were incubated at 30°C with 1 μl ^35^S-methionine (1 000 Ci/mmol, 10 mCi/ml) for 1 h. Labelled proteins were precipitated in TCA, recovered on Whatman glass fibre GF/C filters and subjected to scintillation counting. Metabolic labelling measurements were performed at least in triplicate, and standard error (SE) was calculated. Statistical significance was determined using t-test.

## Results

### SL-RT-ddPCR method enables for detection of small RNA input amounts

Quantitative determination of low levels of small RNAs remains challenging. Because low abundant sdRNAs are not detectable using standard methods, such as northern blot hybridization^23^, we decided to employ an optimized stem-loop reverse transcription (SL-RT) followed by ddPCR. The ddPCR system measures fluorescence intensities of droplets after completion of all thermal cycling. The copy number of target genes is determined based on the number of fluorescent-positive and -negative droplets in a sample well. ddPCR provides an absolute number of RNA copies present in the sample. To define the minimum number of sdRNA copies that can be detected using the pulsed SL-RT-ddPCR method, we spiked 0.5 pg of an exogenous synthetic RNA (37nt in length, no sequence similarity to *S*. *cerevisiae* snoRNAs) to total RNA isolated from *S*. *cerevisiae*. Total RNA was subjected to the SL-RT method. Various dilutions of cDNA were amplified using ddPCR technology. These analyses demonstrated the ability of ddPCR to detect small RNA input levels, as low as 0.005 pg (Suppl. Fig. 1).

### snoRNA and sdRNA levels are dependent upon stress conditions but are independent from each other

Using ddPCR, we investigated the accumulation of individual snoRNAs and sdRNAs across different *S*. *cerevisiae* growth conditions. In the first steps, we have verified the lengths of amplicons with means of standard agarose electrophoresis (Suppl. Fig. 2). Spiked-in synthetic RNA was used as a reference for ddPCR experiments. Absolute concentrations of spike-in reference RNA in different cDNA samples were uniformly distributed, with a mean value of 19,064 (±114) copies/µl. Therefore, we concluded that possible differences in snoRNA or sdRNA concentrations under particular stress conditions would be derived from their abundance and not from biases in experimental design.

**Figure 2:**
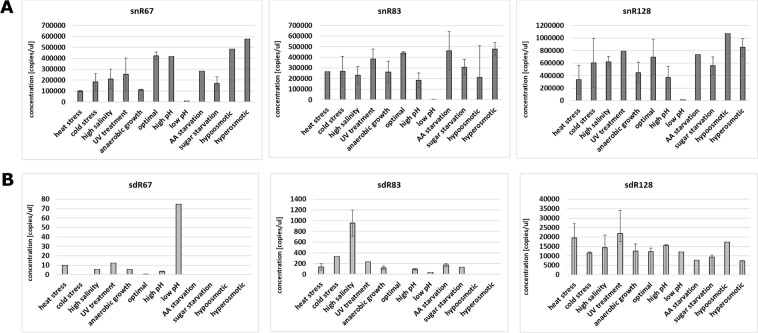
Quantitation of snoRNAs and sdRNAs within the total cellular RNA pool (S30). Concentration (copies/microlitre) of snoRNAs (**A**) and sdRNAs. (**B**) The mean and SE of two experiments are shown. Environmental stress was induced as described in Materials and methods.

All full-length snoRNAs were least abundant under low pH stress, and absolute concentrations were as follows: 10,360 copies/µl for snR67, 717 copies/µl for snR83 and 13,200 copies/µl for snR128 (Fig. 2A and Suppl. Fig. 3A). Except for this stress, where the observed snoRNA concentrations were markedly lower, absolute concentrations under the remaining stress conditions were in a range of 169,000–578,200 copies/µl for snR67, 182,400–463,200 copies/µl for snR83 and 333,900–1,072,000 copies/µl for snR128. Under optimal yeast growth conditions, snR128 was significantly more abundant (698,000 copies/µl) than snR67 and snR83 (422,700 copies/µl and 440,700 copies/µl, respectively).

For sdRNAs, we clearly observed that sdR67 was present in the smallest levels compared to other sdRNAs, just above ddPCR detection level (Fig. 2B and Suppl. Fig. 3B). Its maximum concentration was noted under low pH conditions, at 74 copies/µl. sdR83 was moderately abundant compared to other two sdRNAs. The highest concentration of sdR83 was observed under high salinity conditions, and it reached 953 copies/µl. The most abundant of tested sdRNAs, sdR128, was equally distributed, with the most prominent concentrations under heat stress (19,470 copies/µl), UV shock (21,900 copies/µl) and hypoosmotic stress (17,220 copies/µl).

**Figure 3:**
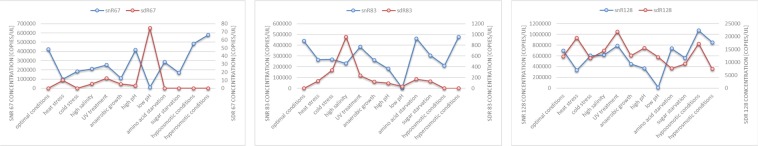
Differential accumulation of snoRNAs and sdRNAs in the cytoplasm. Values are means of replicates that are fully presented in Fig. 2.

Since we observed clear differences in both snoRNA and sdRNA levels across different stress conditions, we next performed analysis of possible correlations between accumulation of these two molecules under particular types of stress (Fig. 3). We observed antagonistic changes under three stress conditions, namely, in low pH stress for snR67, high salinity for snR83 and heat stress for snR128. Under these three conditions, snoRNAs were significantly less abundant and sdRNAs were significantly more abundant. Apart from these observations, in most of the stress conditions, snoRNA levels did not correlate with sdRNA abundance. This observation suggests that differential accumulation of sdRNAs is not directly dependent upon the levels of individual snoRNAs under particular yeast growth conditions. This suggests possible stress-dependent regulation of sdRNA excision.

### snoRNAs and sdRNAs associate with ribosomes *in vivo* in a stress-dependent manner

**Figure 4:**
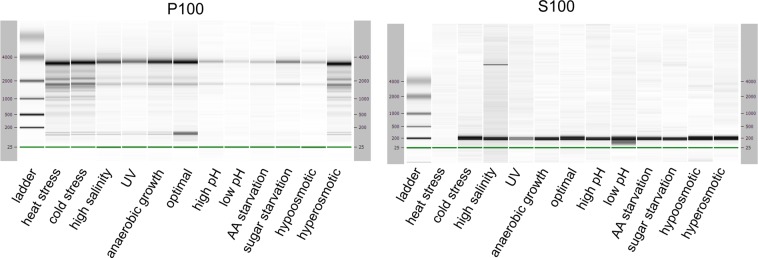
RNA length composition of the P100 (ribosome-enriched pellet) and S100 (ribosome-depleted supernatant) fractions derived from the lysates of native and stressed cells. RNA was isolated with TRI Reagent and subjected to Agilent RNA 6000 Nano assay. The bands corresponding to ribosomal RNAs (18 S rRNA of ~2000 bp and 26 S rRNA of ~3,800 bp) are visible in P100 fraction. Low molecular weight RNAs, up to 200 bp are mostly present in S100 fraction.

Ultracentrifugation of yeast lysates allowed us to separate ribosome-containing pellet (P100) from post-ribosomal supernatant (S100) and to verify the cellular distribution of sdRNAs between these two fractions. The purity of P100 and S100 fraction was verified with Agilent Bioanalyzer 2100 with the use of RNA Nano 6000 kit (Fig. 4). The activity of the ribosomes within P100 pellet was verified using translation of poly(U) template *in vitro* (Suppl. Fig. 4).

For accurate quantification of snoRNAs and sdRNAs within P100 and S100 fractions, we employed ddPCR technology. Spiked-in synthetic RNA was used as a control for experiments, as previously described for total cellular RNA pools. Absolute concentrations of spike-in reference RNA in different cDNA samples derived from ribosome-associated RNA pools were uniformly distributed, with a mean value of 7,573 (±11) copies/µl in P100 and 20,125 (±122) copies/µl in S100 fraction. Therefore, we concluded that possible differences in snoRNA or sdRNA concentrations in different pools derive from their differential association with ribosomes and not from biases due to experimental design.

**Figure 5:**
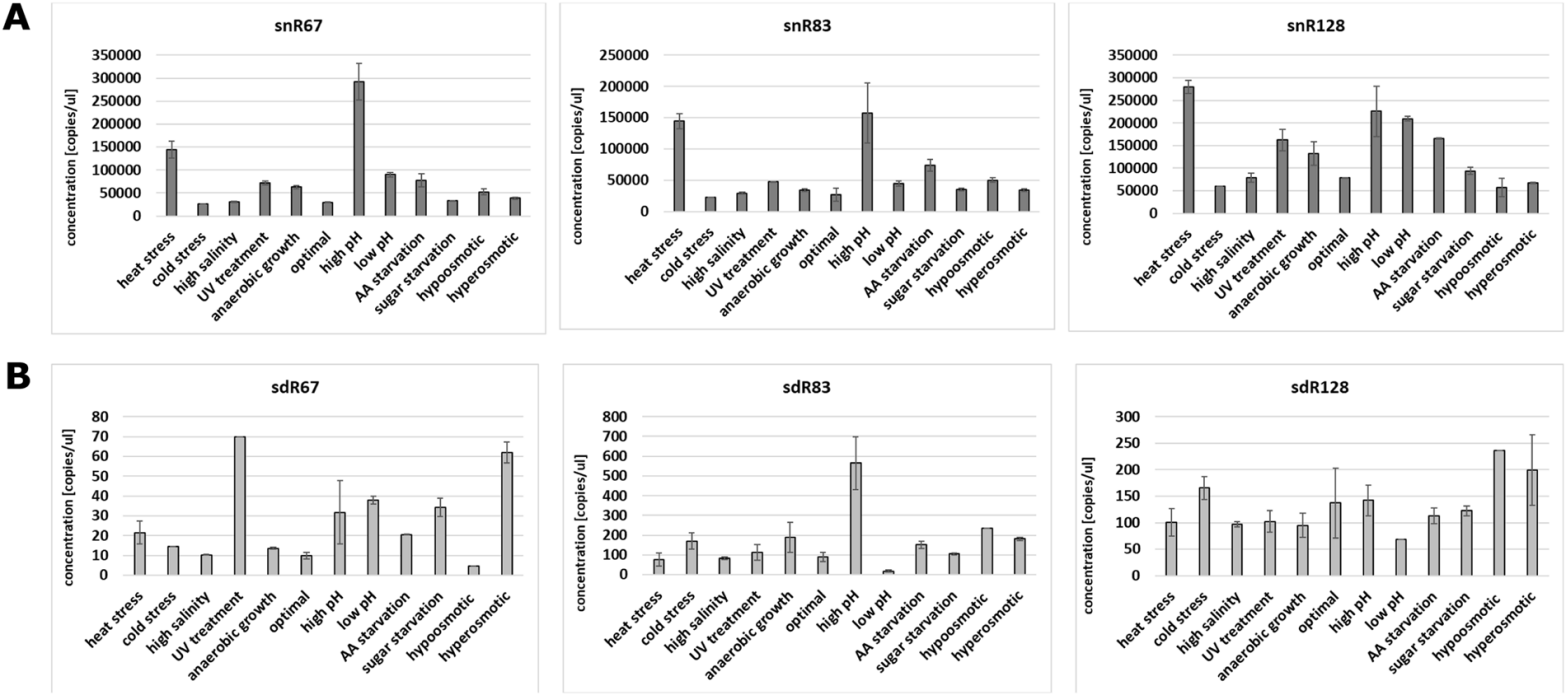
Quantitation of snoRNAs and sdRNAs within ribosome-associated RNAs (P100). Concentration (copies/microlitre) of snoRNAs (**A**) and sdRNAs (**B**) is presented. The mean and SE of two experiments are shown. Environmental stress was induced as described in Materials and methods.

The first observation was that both full-length snoRNAs and sdRNAs are present in ribosome-containing fractions (Fig. 5 and Suppl. Fig. 5). Moreover, analysis of snoRNA sdRNA concentrations in P100 fraction obtained from yeast cultured under 12 different growth conditions illustrate that this association is strongly stress-dependent. snoRNAs are present on considerable quantities in ribosome-containing fractions (Fig. 5A), exceeding the concentration of sdRNAs over 800 times on average (Fig. 5B). In case of snR67, its highest concentration in ribosomal fractions was observed when ribosomes were isolated from yeast subjected to high pH conditions (292,400 copies/µl). The lowest snR67 concentration was noted in optimal conditions, as well as cold stress, and it oscillated approximately 2,800 copies/µl. snR83 was characterized by the lowest concentration among all three examined snoRNAs, with the maximum of 157,200 copies/µl in high pH conditions. Similarly, to snR67, cold stress caused the lowest accumulation of snR83 in the ribosomal fraction (22,100 copies/µl). The highest concentration in ribosomal fraction was observed for snR128, ranging from 57,300 copies/µl (hypoosmotic growth conditions) to 279,400 copies/µl (heat shock). In general, heat stress and high pH stress induced significant increases in ribosome-associated snoRNAs.

Among investigated sdRNAs (Fig. 5B), sdR67 was the least abundant, reaching a maximum of 70 and 62 copies/µl after UV treatment and hyperosmotic stress conditions, respectively. The lowest accumulation of sdR67 in the ribosomal fraction was detected during hypoosmotic stress with 4.5 copies/µl. Generally, the amount of sdR83 was significantly higher in most stress conditions compared to sdR67. The highest concentration was observed during high pH conditions (565.5 copies/µl). In contrast, in low pH conditions, only 18.5 copies/µl of sdR83 were detected. For sdR128, a variety of growth conditions did not strongly affect its presence in the ribosome-associated RNA pool. Highest sdR28 accumulation was observed in hypo- and hyperosmotic conditions, reaching 236 and 199 copies/µl, respectively.

**Figure 6:**
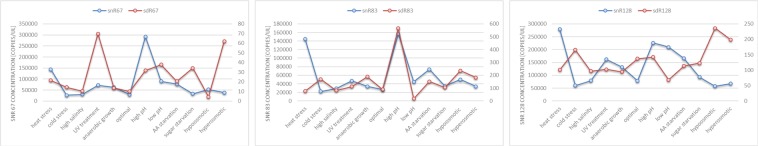
Differential accumulation of snoRNAs and sdRNAs in ribosomes. Values are means of replicates that are fully presented in Fig. 5.

To elaborate on possible *in vivo* interactions with ribosomes, we compared the differential accumulation of both snoRNAs and sdRNAs in ribosome-associated RNA fractions (Fig. 6). In cases of high pH stress, both snR83 and its derivative, sdR83, were highly abundant. Except for this case, in the remaining stress conditions, snoRNA and sdRNA levels within ribosome-associated RNA pools were not well correlated. Such observation suggests that stress-dependent association of full-length snoRNAs and small sdRNAs with yeast ribosomes is independent.

**Figure 7:**
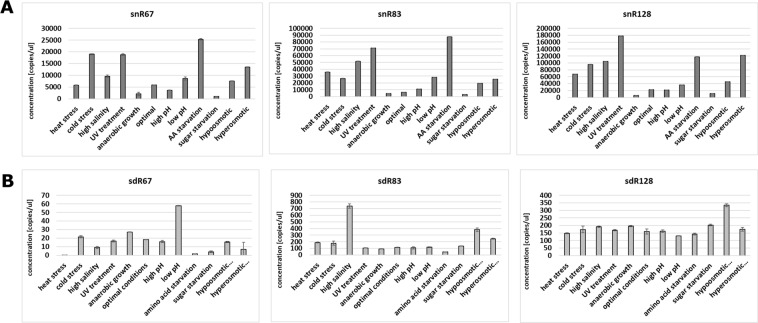
Quantitation of snoRNAs and sdRNAs within post-ribosomal supernatant. Concentration (copies/microlitre) of snoRNAs (**A**) and sdRNAs. (**B**) The mean and SE of two experiments are shown. Environmental stress was induced as described in Materials and methods.

Both full-length snoRNAs and sdRNAs are present in post-ribosomal supernatant fractions (Fig. 7A and Suppl. Fig. 6A) and their concentration differs in yeast cultivated under different conditions. snoRNA concentrations are much more higher than sdRNA concentrations, similarly like in S30 and P100 fractions. In contrast to the situation observed in S30 fractions, snoRNAs were not least abundant under low pH stress but under sugar starvation (1,018 copies/µl for snR67, 4,510 copies/µl for snR83 and 6,070 copies/µl for snR128) and anaerobic growth (2,060 copies/µl for snR67, 3,115 copies/µl for snR83 and 11,650 copies/µl for snR128). Except of these conditions, absolute concentrations of snoRNAs in S100 fraction were in a range of 5,880–25,350 copies/µl for snR67, 11,020–87,700 copies/µl for snR83 and 22,200–121,850 copies/µl for snR128.

For sdRNAs, we clearly observed that sdR67 was present in the smallest levels compared to other sdRNAs, just above ddPCR detection level (Fig. 7B and Suppl. Fig. 6B). Its maximum concentration was noted under low pH conditions, at 57 copies/µl. The highest concentration of sdR83 was observed under high salinity conditions, and it reached 738 copies/µl. sdR128, was equally distributed (147–202 copies/µl), with the exception of hypoosmotic stress (334 copies/µl).

**Figure 8:**
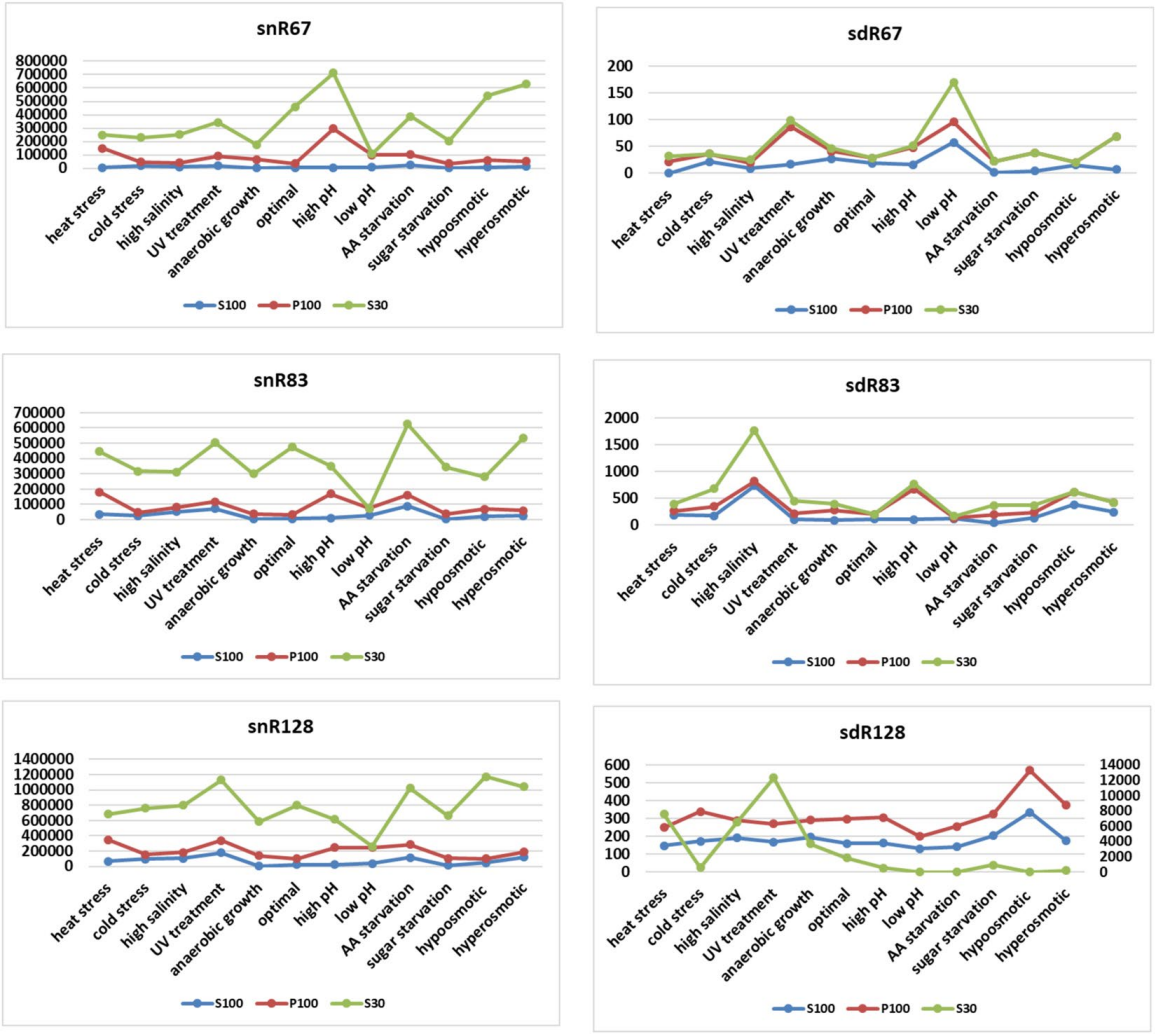
Differential accumulation of snoRNAs and sdRNAs in S30, S100 and P100 fractions. Values are means of replicates that are fully presented in Figs. 2, 5 and 7.

We next performed analysis of possible correlations between accumulation of snoRNAs and sdRNAs under particular types of stress in all cellular fractions analyzed (Fig. 8). We observed that in all cases an absolute concentration of both, snoRNAs and sdRNAs was higher in S30 than in P100 and S100 fractions, as expected. Patterns of snR and sdR accumulation in ribosome-containing fractions resembles those in post-ribosomal supernatant rather than total cellular RNA pool. This suggest that stress-related differential accumulation of snoRNAs and sdRNAs in ribosome fractions is related to their possible functional interactions with the ribosomes. Concentrations of snR and sdR in S100 fractions were lower than in P100 and S30, which implicates that a prominent portion of snoRNAs and sdRNAs present in the cell associate with the ribosomes. This suggests possible stress-dependent regulation of ribosome function by snoRNAs and/or sdRNAs.

### Yeast sdRNAs inhibit translation *in vitro* and *in vivo*

**Figure 9:**
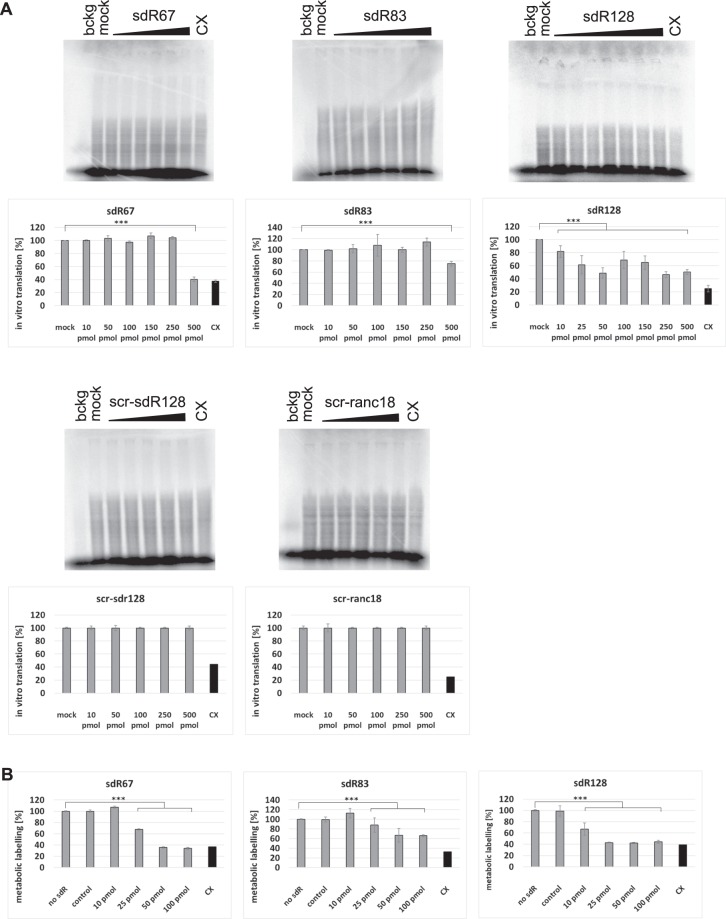
S. cerevisiae snoRNA-derived RNAs inhibit protein biosynthesis. Cycloheximide (CX) served as a control translational inhibitor. The mean and SE of three to six experiments are shown. ***p-value < 0.005. (**A**) Dose-dependent effects of sdRNAs on *in vitro* translation system. The autoradiographs of representative SDS polyacrylamide gels of *in vitro* translation assays performed in the absence (mock) or in the presence of synthetic sdRNAs, are shown. Translational efficiency of endogenous yeast mRNA is shown on graphs, as a percentage [%] of activity of the control experiment without sdRNA. (**B**) Incorporation of ^35^S-methionine into the translatome of yeast spheroplasts is presented as translation *in vivo* efficiency [%]. The efficiency of metabolic labelling in the absence of sdRNA was set at 100%, and spheroplasts harbouring synthetic sdRNAs were compared to this value.

The observation that sdRNAs accumulate in ribosomal fractions in a stress-dependent manner led to speculation of their potential function as regulatory ncRNAs during protein biosynthesis. To clarify this, we set up an *in vitro* translation system for *S*. *cerevisiae* grown under optimal conditions using the total endogenous mRNA pool as template and ^35^S-methionine incorporation into proteins as readout (Fig. 9A). In the presence of the ribosome-targeting antibiotic cycloheximide, all radiolabeled bands were drastically reduced, demonstrating that the ^35^S-methionine labeling of proteins was translation-dependent. When the assay was performed in the presence of synthetic sdR67, sdR83 or sdR128 we observed a reproducible inhibitory effect on translation. Introduction of 35.7 µM sdR67 or sdR83 (equivalent to 500 pmol per reaction) reduced *in vitro* translation to 40% and 75%, respectively. The highest inhibitory effect was observed for sdR128. Even small concentrations of sdR128, such as 0.7 µM (equivalent to 10 pmol per reaction), observably reduced *in vitro* protein synthesis. Conversely, the addition of the highest tested dose of the scrambled sdRNA128 (scr-sdR128) or 18nt-long control RNA oligomer (scr-ranc18), 35.7 µM, did not have any influence on translation efficiency.

To investigate whether *in vitro* effects have a physiological significance in yeast we used electroporation to introduce synthetic sdRNAs into *S*. *cerevisiae* cells. Conditions for small RNA electroporation were optimized in our previous studies, where quantification of the uptake efficiency of the synthetic 18-mer into spheroplasts indicated the presence of about 200,000 molecules per cell, thus roughly equaling the ribosome concentration^22^. We measured ^35^S-Met incorporation into newly synthetized proteins in the presence and absence of sdR128, sdR83 and sdR67. As controls, we have used RNA oligomers with similar length to tested sdRNAs: scr-sdR128 (22 nt) as a control for sdR128, scr-ranc18 (18 nt) for sdR67 and spike-in RNA (44 nt) for sdR83. Control oligomers did not affect *in vivo* translation (Fig. 9B). Yeast sdRNAs decreased translational efficiency *in vivo*. Inhibition efficiency was in a range similar to the well-known ribosome-targeting antibiotic cycloheximide. Similarly to *in vitro* translation, sdR128 had the highest inhibitory effect on *in vivo* translation.

**Figure 10:**
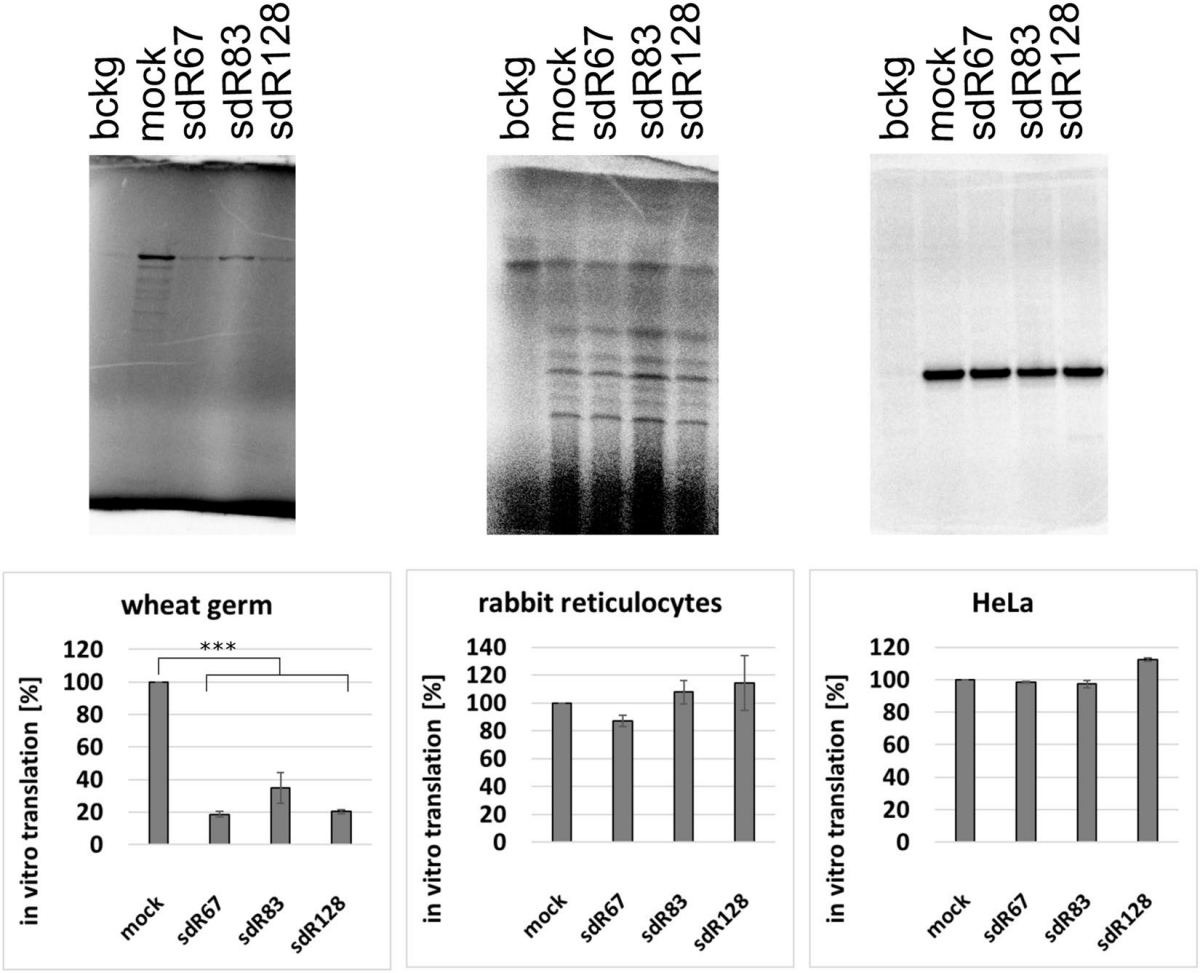
S. cerevisiae sdRNAs inhibition of protein biosynthesis in *in vitro* eukaryotic translation systems. A representative *in vitro* translation of synthetic non-capped poly(A)-tailed luciferase mRNA in wheat germ extracts, rabbit reticulocyte lysates and HeLa cell lysates. The reactions were performed in the absence (mock) or presence of yeast sdRNAs (500 pmol). The mean and SE of three *in vitro* translation experiments are shown beneath the gels. Cropped gels are displayed, full-length gels are included on Suppl. Fig. 7. ***p-value < 0.005

Because both snoRNAs and ribosomes are universally conserved, we tested if sdRNA-mediated repression of translation is functionally conserved in other eukaryotic species as well. To test this possibility, we examined three cell-free *in vitro* translation systems using wheat germ extracts, rabbit reticulocyte lysates, and HeLa cell lysates (Fig. 10). *In vitro* translation reactions were performed either in the absence (mock) or in the presence of synthetic sdRNAs. The addition of *S*. *cerevisiae* sdRNAs reproducibly inhibited *in vitro* protein biosynthesis in the wheat germ system (Fig. 10). *In vitro* translation was very mildly inhibited by yeast sdR67 in the rabbit reticulocyte but not by sdR83 nor sdR128. No inhibition was observed in human systems. These data suggest that *S*. *cerevisiae* sdRNAs might potentially inhibit some translation systems in selected eukaryotes.

## Discussion

In a recent study, we revealed that snoRNAs and sdRNAs in *Saccharomyces cerevisiae* are present in the cytoplasm, where they associate with ribosomes^30^. Herein, we show that the presence of *S*. *cerevisiae* sdRNA in the ribosomal fractions influences protein biosynthesis *in vivo* and *in vitro*. Moreover, accumulation of snoRNAs and sdRNAs in the cytoplasmatic and ribosomal fractions is strongly dependent upon stress conditions. For the first time, we have shown that snoRNA and sdRNA levels in the cytoplasm and their possible association with ribosomes are independent from each other.

It has been already shown that snoRNAs play crucial roles in adaptation to stress conditions^21^. In yeast, U3 snoRNA (snR17) is upregulated during heat shock, amino acid starvation and sugar starvation and downregulated under high salinity and hyperosmotic conditions^31^. Interestingly, the expression pattern of snoRNAs studied herein do not resemble those of snR17. In Chinese hamster ovary (CHO) cells U14 snoRNA, which corresponds to yeast snR128, is strongly upregulated during heat shock^19^. In yeast, however, we observed almost 2-fold downregulation of snR128 during heat stress. Such observations suggest that different snoRNAs possess distinct expression patterns during stress responses, which might be related to their functions.

Despite the fact that dysregulation of snoRNAs is involved in adaptation to stress conditions, only a few publications have reported the roles of sdRNAs in tumour development, which may be considered a stress for human cells^21,32,33^. Expression analysis of sdRNAs has been performed in several types of cancer with the conclusion that accumulation of sdRNAs is associated with malignant transformation and that increased global production or accumulation of sdRNAs is already occurring in the early stages of cancer. Surprisingly, there is no data reporting sdRNA levels in canonical stress conditions. To our knowledge, differential expression of sdRNAs has not previously been reported. Here, we report for the first time that sdRNAs in *S*. *cerevisiae* are present under a wide repertoire of growth conditions, though in some cases in limited amounts. So far, sdRNAs have been reported to localize in the cytoplasm in organisms where they act within microRNA pathways^6,10,12–14^. Here, we present new data demonstrating cytoplasmatic localization of sdRNAs in an organism that lacks miRNA pathways.

Moreover, for the first time, we present data showing that snoRNAs and sdRNAs are differentially abundant in both the cytoplasmatic and in ribosomal fractions. Such observations indicate that sdRNAs and snoRNAs might perform distinct cellular functions in response to stress conditions, probably during translation regulation. These observations strongly support the hypothesis for separate roles of both snoRNAs and sdRNAs during stress conditions.

The presence of both sdRNAs and snoRNAs in ribosome-associated RNAs indicates the possible existence of a novel, yet to be discovered stress-dependent translation regulation mechanism. The data presented herein strongly suggest that possible interactions between yeast ribosomes and sdRNAs downregulate translational activity during optimal growth conditions. Moreover, the observation of the inhibition of protein synthesis by yeast sdRNAs in the wheat germ system suggests the mechanism of translation regulation by sdRNAs is evolutionarily conserved. This suggests that the mode of action and, thus, also the ribosomal target site, is conserved in a range of eukaryotic species. In this aspect, sdRNAs could be classified as an example of ribosome-associated noncoding RNAs (rancRNAs), next to an mRNA exon-derived 18-residue-long ncRNA^22^ and tRNA-derived fragments^26^ previously described by our lab.

## Supplementary information

Supplementary information
