# Supplementary material for: Levels of sdRNAs in cytoplasm and their association with ribosomes are dependent upon stress conditions but independent from snoRNA expression
Source: Sci Rep. 2019 Dec 5;9:18397. doi: 10.1038/s41598-019-54924-2 (PMC6895083; doi:10.1038/s41598-019-54924-2)
Supplement: Supplementary file 1 — Supplementary information [file 41598_2019_54924_MOESM1_ESM.pdf]

Supplementary material

Levels of sdRNAs in cytoplasm and their association with ribosomes are dependent upon stress conditions but independent from snoRNA expression

Anna M. Mleczko[#], Piotr Machtel[#], Mateusz Walkowiak, Anna Wasilewska, Piotr J. Pietras, Kamilla Bąkowska-Żywicka*

Institute of Bioorganic Chemistry Polish Academy of Sciences, Noskowskiego 12/14, 61-704 Poznań, Poland

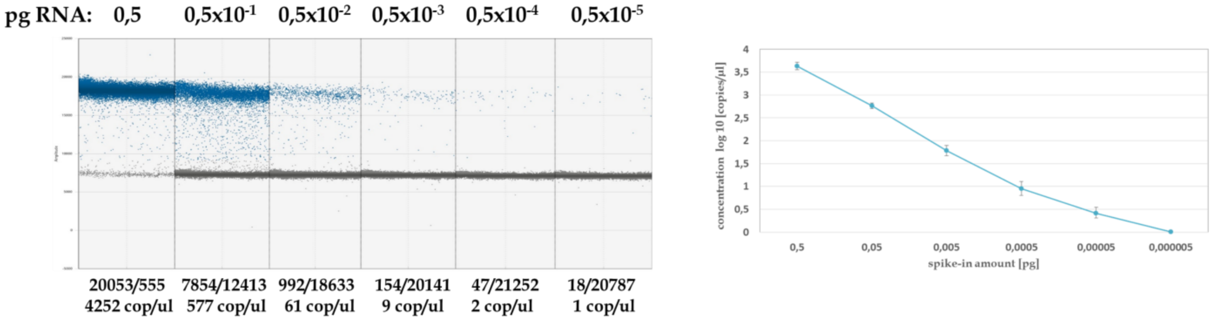

Supplementary Figure 1. Detection and quantitation of synthetic spike-in RNA.
A) Fluorescence amplitude of different concentrations of spike-in RNA. Levels of input RNA are reported at the top, whereas the number of positive/negative droplets and the concentration (copies/microlitre) are reported on the bottom of each panel.
B) The relationship between calculated copies/microlitre and input RNA.

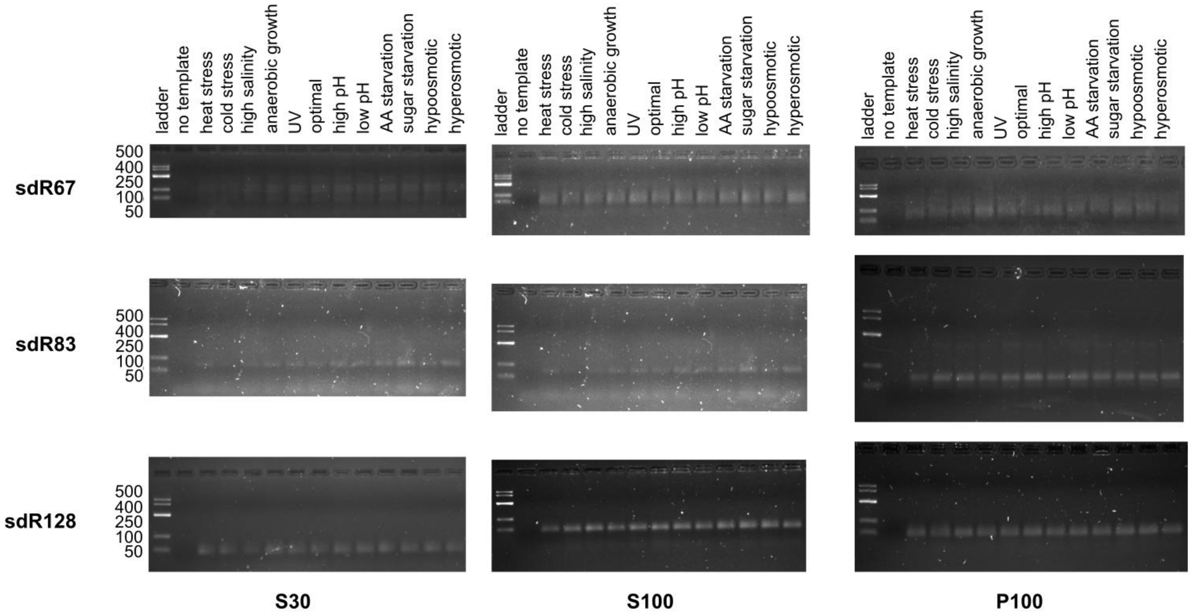

Supplementary Figure 2. Detection of sdRNAs within total cellular RNA (S30), postribosomal supernatant (S100) and ribosomal pellet (P100) pools.
Stem-loop RT-PCR analyses of expression of sdRNAs. 100 ng of S30, P100 or S100 RNA was used for reverse transcription reactions. 40 PCR cycles were performed. The band corresponds to the amplification product of sdRNAs.

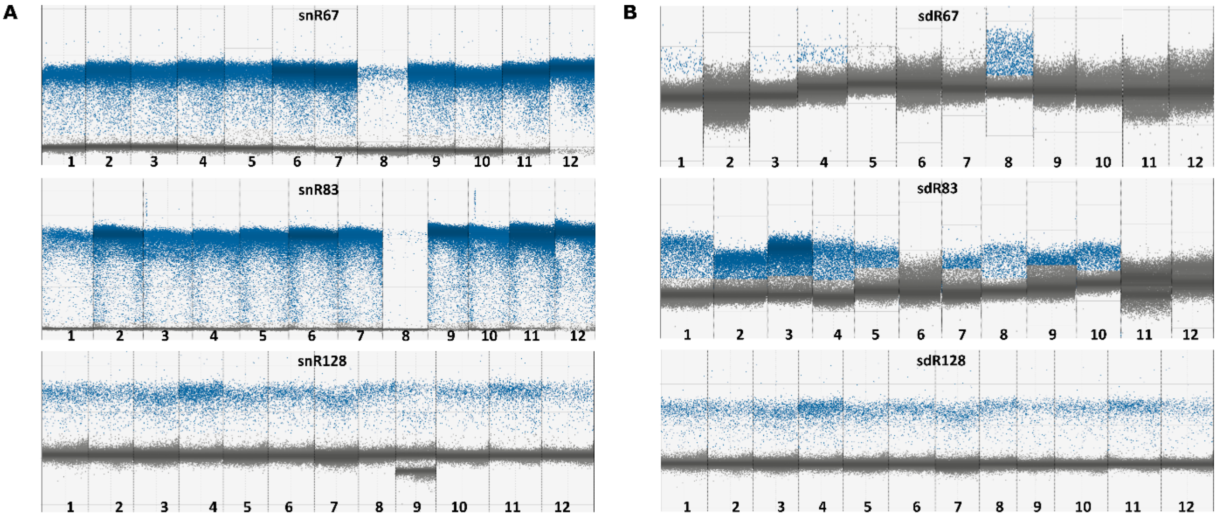

Supplementary Figure 3. Detection of snoRNAs and sdRNAs within total cellular RNA pools.

Representative fluorescence amplitude of snoRNAs (A) and sdRNAs (B). Positive droplets are in shown in blue, negative droplets in grey. Slot numbers correspond to stress conditions as follows: 1 – heat stress, 2 – cold stress, 3 – high salinity, 4 – UV treatment, 5 – anaerobic growth, 6 – optimal conditions, 7 – high pH, 8 – low pH, 9 – AA starvation, 10 – sugar starvation, 11 – hypoosmotic conditions, and 12 – hyperosmotic conditions.

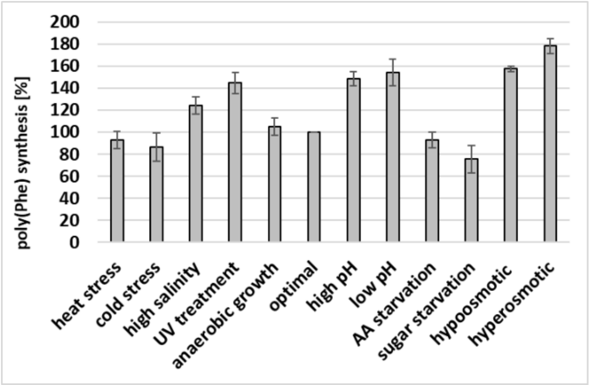

Supplementary Figure 4. Activity of ribosomes isolated from yeast cultured under different conditions.

Activity of the ribosomes was measured as translation of poly(U) templates in vitro. Activity of the ribosomes isolated from yeast grown under optimal conditions was set to 100%.

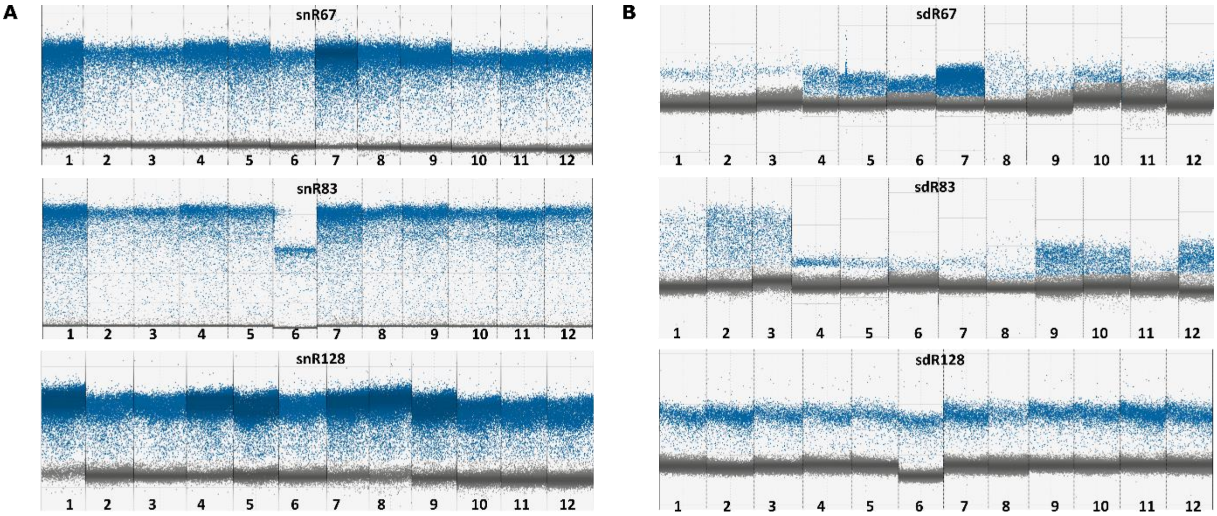

Supplementary Figure 5. Detection of snoRNAs and sdRNAs within ribosome-associated RNAs.

Representative fluorescence amplitude of snoRNAs (A) and sdRNAs (B). Positive droplets are in shown blue, negative droplets in grey. Slot numbers correspond to stress conditions as follows: 1 – heat stress, 2 – cold stress, 3 – high salinity, 4 – UV treatment, 5 – anaerobic growth, 6 – optimal conditions, 7 – high pH, 8 – low pH, 9 – AA starvation, 10 – sugar starvation, 11 – hypoosmotic conditions, and 12 – hyperosmotic conditions.

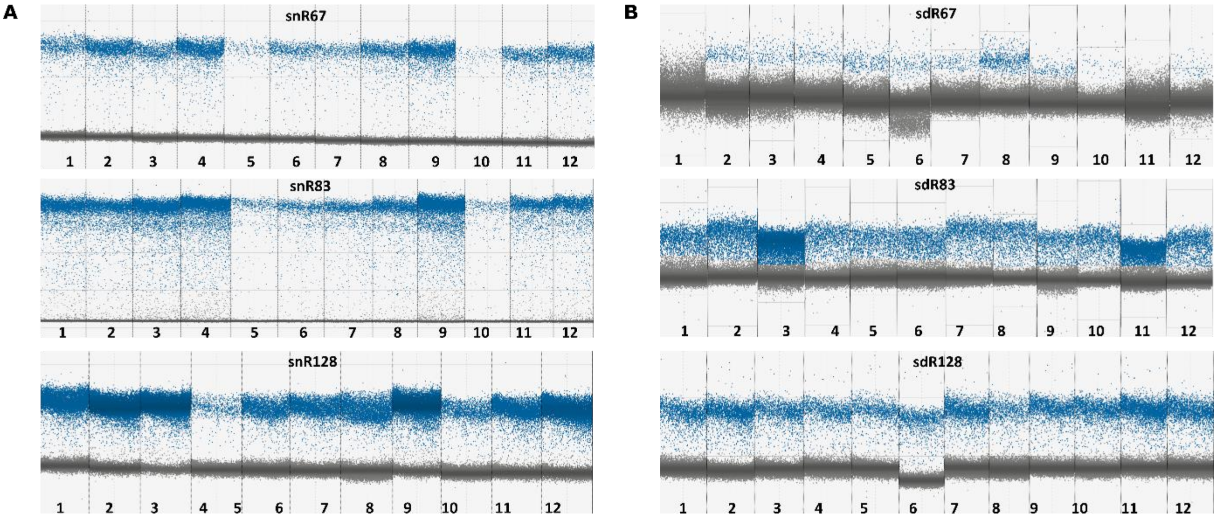

Supplementary Figure 6. Detection of snoRNAs and sdRNAs within the post-ribosomal supernatant fraction (S100).

Representative fluorescence amplitude of snoRNAs (A) and sdRNAs (B) is presented. Positive droplets are in blue, negative droplets – in grey. Numbers correspond to stress conditions: 1 – heat stress, 2 – cold stress, 3 – high salinity, 4 – UV treatment, 5 – anaerobic growth, 6 – optimal conditions, 7 – high pH, 8 – low pH, 9 – AA starvation, 10 – sugar starvation, 11 – hypoosmotic conditions, 12 – hyperosmotic conditions.

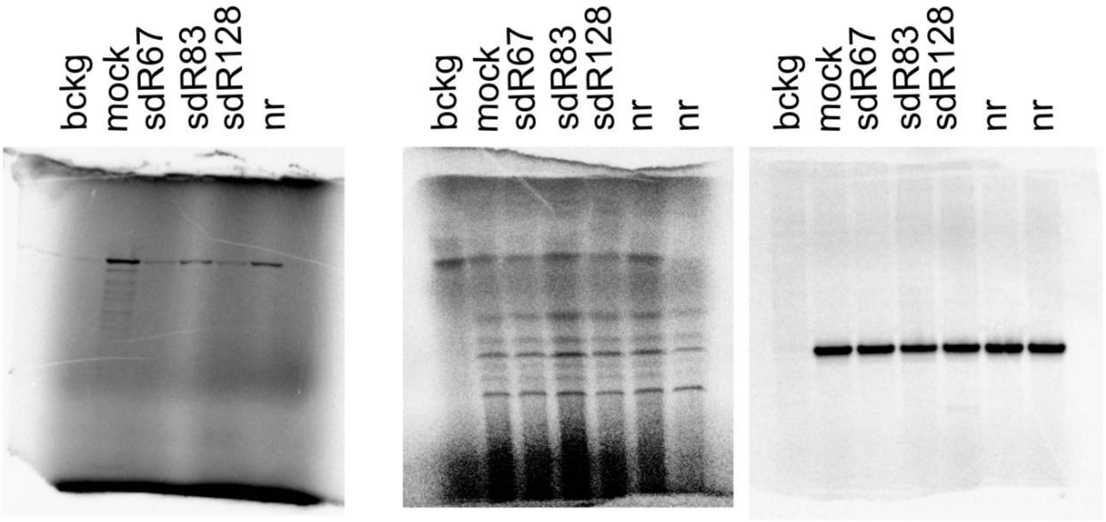

Supplementary Figure 7. *S. cerevisiae* sdRNAs inhibition of protein biosynthesis in *in vitro* eukaryotic translation systems.
A representative of full-length gels displaying in vitro translation in wheat germ extracts, rabbit reticulocyte lysates and HeLa cell lysates. The reactions were performed in the absence (mock) or presence of yeast sdRNAs (500 pmol). nr - RNA oligomer not relevant to the present study. Cropped gels and full description are presented on Fig. 10.
